# Evaluating the surgical trainee ergonomic experience during minimally invasive abdominal surgery (ESTEEMA study)

**DOI:** 10.1038/s41598-024-63516-8

**Published:** 2024-05-31

**Authors:** Cassandra Chan, Ying Ching Tan, Ee Wen Lim, Jin-Yao Teo, Jinlin Lin, Winson JianHong Tan, Gerald Ci An Tay, Emile Kwong-Wei Tan, Isaac Seow-En

**Affiliations:** 1 Duke-NUS Medical School, 8 College Rd, Singapore, 169857 Singapore; 2 Department of Colorectal Surgery, Singapore General Hospital, Outram Road, Singapore, 169608 Singapore; 3 Department of Hepatopancreatobiliary and Transplant Surgery, Singapore General Hospital, Outram Road, Singapore, 169608 Singapore; 4 Department of General Surgery, Changi General Hospital, 2 Simei Street 3, Singapore, 529889 Singapore; 5 Department of General Surgery, Sengkang General Hospital, 110 Sengkang East Way, Singapore, 544886 Singapore; 6 Department of Head and Neck Surgery, Singapore General Hospital, Outram Road, Singapore, 169608 Singapore

**Keywords:** Occupational health, Gastrointestinal system

## Abstract

Minimally invasive abdominal surgery (MAS) can exert a physical cost. Surgical trainees spend years assisting minimally-invasive surgeries, increasing the risk of workplace injury. This prospective questionnaire-based cohort study was conducted amongst general surgery residents in Singapore. Residents assisting major MAS surgery were invited to complete anonymous online survey forms after surgery. The Phase 1 survey assessed physical discomfort scores and risk factors. Intraoperative measures to improve ergonomics were administered and evaluated in Phase 2. During Phase 1 (October 2021 to April 2022), physical discomfort was reported in at least one body part in 82.6% (n = 38) of respondents. Over a third of respondents reported severe discomfort in at least one body part (n = 17, 37.0%). Extremes of height, training seniority, longer surgical duration and operative complexity were significant risk factors for greater physical discomfort. In Phase 2 (October 2022 to February 2023), the overall rate of physical symptoms and severe discomfort improved to 81.3% (n = 52) and 34.4% (n = 22) respectively. The ergonomic measure most found useful was having separate television monitors for the primary surgeon and assistants, followed by intraoperative feedback on television monitor angle or position. Close to 20% of survey respondents felt that surgeon education was likely to improve physical discomfort.

## Introduction

The current prevalence and widespread acceptance of minimally invasive surgery has been more than 200 years in the making, comprising numerous scientific breakthroughs along the way, via collaboration amongst clinicians, biomedical innovators and industry partners^1^. Today, laparoscopy has been evaluated for virtually every thoracoabdominal organ system and can be considered the gold standard approach for appendectomy^2^, cholecystectomy^3^, and colectomy^4^, amongst others.

The advantages of minimally invasive abdominal surgery (MAS) are well documented, including reduced postoperative pain and wound complications, improved recovery, quicker hospital discharge and better cosmesis. For the surgeon, enhanced operative visualization with the modern laparoscope and high-definition television monitor system allows for more precise dissection, decreasing the risk of unintended tissue injury and bleeding. These benefits drive patient demand and operator interest, shaping surgical training and fostering the use of MAS for increasingly complex conditions. Ongoing innovation has encouraged the introduction of novel MAS techniques including robotic, endoscopic, single incision, and natural orifice surgery.

MAS is not without its challenges. Surmounting the steep learning curves of MAS^5,6^ requires formal training and structured proctorship programs^7^. Over the recent decades, the proportion of minimally invasive compared to open procedures performed by surgical trainees has risen substantially^8–10^. Accordingly, surgical trainees or residents spend a considerable period of their training assisting MAS procedures as well.

The physical cost of MAS surgery to the operating surgeon is well documented. Complex tissue manipulation using laparoscopic techniques requires significantly more upper-extremity muscle effort compared to open surgery^11^, increasing the risk of digital nerve injury^12^, while lengthy maintenance of static positions predisposes the MAS surgeon to musculoskeletal disorders^13^. The operating room has been described as a “hostile environment” due to poor ergonomic conditions for laparoscopic surgeons^14^, with Park et al. demonstrating an 87% incidence of physical discomfort amongst minimally invasive operators^15^. The spectrum of injuries associated with minimal access surgery has been collectively termed MAS-related surgeon morbidity syndrome^16^.

The ergonomic impact of MAS surgery to the surgical assistant is less well understood. Like the surgeon, operative assistants also experience significant physical discomfort^17,18^, which may be worse than that of the primary surgeon^19^. Surgical trainees spend years assisting laparoscopy and other minimally invasive surgeries, increasing the risk of sustaining workplace injuries, with a negative influence on job satisfaction and career longevity.

While several reviews and recommendations exist to improve surgeon ergonomics during MAS surgery^20–22^, similar guidelines are lacking for surgical assistants. We aimed to assess the incidence of and risk factors for physical discomfort amongst surgical trainees assisting MAS surgery, as well as evaluate methods used to improve assistant well-being.

## Materials and methods

This prospective questionnaire-based cohort study was conducted amongst general surgery residents (trainees) from Singapore Health Services (SingHealth) General Surgery Residency Program. These residents rotated across three general hospitals: Singapore General Hospital, Changi General Hospital, and Sengkang General Hospital, within Singapore’s largest healthcare cluster.

The study was administered in 2 phases. Prior to commencement of Phase 1, study objectives and methodology for both Phases 1 and 2 were explained to all general surgery residents. Informed consent for study participation was obtained from all residents. Attending surgeons were only informed of the study prior to Phase 2. Residents assisting in major minimally invasive upper gastrointestinal, hepatopancreatobiliary, or colorectal surgery from the three SingHealth hospitals were invited to complete anonymous online survey forms immediately after surgery. A major surgery was defined by the Ministry of Health Singapore Table of Surgical Procedures Table Code 4A and above^23^. Residents were informed to limit their survey responses to two for Phase 1 and three for Phase 2 (up to a maximum of five responses per individual).

Phase 1 was conducted from 1 October 2021 to 1 April 2022. The Phase 1 survey consisted of 13 questions, including resident and surgery characteristics, physical discomfort scores for each body part, risk factors for physical discomfort and suggestions for ergonomic improvement (Table 1). Physical symptom scores were indicated by a 5-point Likert scale. The options provided for factors contributing to physical discomfort and suggestions for future ergonomic improvement (6 options each, as well as a free text option), were previously determined by a team of chief residents in their 4th and 5th (final) year of residency training, and one minimally invasive attending surgeon from the upper gastrointestinal, hepatopancreatobiliary, and colorectal subspecialties. Multiple responses were allowed per resident for Questions 12 and 13.

**Table 1:** ESTEEMA Phase 1 and 2 surveys of SingHealth general surgery residents following minimally invasive abdominal surgery (MAS).

Question	Options
Resident characteristics	
1.Gender	Male/Female
2.Height	(cm)
3.Dominant hand	Right/Left
4.Glove size	5.5/ 6/ 6.5/ 7/ 7.5/ 8
5.Year of residency training	Year 1/2/3/4/5
Surgery characteristics	
6.Surgical role	Cameraman/Primary assistant
7.Conventional laparoscopy or robotic laparoscopy	Laparoscopic/Robotic
8.Surgical duration	(hours)
9.Brief description of surgical procedure e.g., anterior resection, gastrectomy	Free text
10.Complexity of surgery (perceived)	Routine/Complex
Physical symptoms	
11.Degree of physical discomfort (i.e., numbness, stiffness, fatigue, or pain) in each body part during or immediately following surgery (1 = no discomfort; 5 = maximum discomfort)	Eyes – 1/2/3/4/5 Neck – 1/2/3/4/5 Shoulder/Arm – 1/2/3/4/5 Wrist/Hand/Finger – 1/2/3/4/5 Trunk – 1/2/3/4/5 Back – 1/2/3/4/5 Leg – 1/2/3/4/5 Foot – 1/2/3/4/5
Perceived risk factors (Phase 1 only)	
12.Factor(s) contributing to physical discomfort (current surgery) Note: not limited to one risk factor	A. Restricted posture B. Uncomfortable table height/position C. Procedural difficulties D. Long surgical duration E. Height disparity with operating surgeon F. Television monitor angle/position G. Others (Free text)
Suggested measures for ergonomic improvement (Phase 1 only)	
13.Measure(s) which may be useful to reduce surgical assistant physical discomfort (for future surgeries of the same type) Note: not limited to one suggestion	A. Separate monitors for surgeon and assistants B. Allowing assistant to sit on adjustable chair C. Intraop feedback on table height/position D. Intraop feedback on monitor angle/position E. Intraop feedback on positional changes F. 5-min break every 1–2 h of surgery G. Others (Free text)
Evaluation of ergonomic improvement measures (Phase 2 only)	
A. Separate monitors for surgeon and assistant B. Allowing assistant to sit on adjustable chair C. Intraop feedback on table height/position D. Intraop feedback on monitor angle/position E. Intraop feedback on positional changes F. 5-min break every 1–2 h of surgery	1. Evaluated and useful 2. Evaluated and not useful 3. Not evaluated Note: each measure was individually assessed

Phase 2 of the study was conducted from 1 October 2022 to 1 February 2023. Prior to this phase, attending surgeons who routinely performed minimally invasive abdominal surgery from the three participating institutions were informed about the study via email and invited to administer the suggestions to improve assistant ergonomics during major MAS. Residents were encouraged to approach these surgeons preoperatively to remind them to institute these measures during the surgery, when possible. The Phase 2 survey included the first 11 questions from the Phase 1 survey, as well as an additional question assessing residents’ perceived usefulness of the ergonomic improvement measures listed from Phase 1 (Table 1).

Data was analyzed using the Statistical Package for Social Sciences (SPSS), version 28.01. The Mann–Whitney U test or Kruskal–Wallis H test was used for analysis of variables. Statistical significance was set at two-tailed *p* value < 0.05.

This study was registered as a quality improvement project with Singapore General Hospital (1388FY2110).

### Ethics approval

This research conformed to the provisions of the Declaration of Helsinki. Approval for the study was waived by the SingHealth Institutional Review Board. Informed consent for study participation was obtained from all study participants. This study was registered as a quality improvement project with Singapore General Hospital (1388FY2110).

## Results

During the study period, a total of 32 residents were enrolled in the SingHealth General Surgery Residency Program, with seven residents in each batch from Year 1 to Year 3, six residents in Year 4 and five residents in Year 5. Most of the Year 3 program consists of elective postings and non-general surgery rotations, including cardiothoracic surgery, anesthesia and intensive care rotations, as well as pediatric surgery.

### Phase 1 results

Forty-six completed surveys were received during Phase 1 of the study. Physical discomfort scores during MAS surgery across all body parts were summated (maximum score 40, minimum score 8) for each survey response. Physical discomfort was reported in at least one body part (discomfort score 2 in ≥ 1 area) in 82.6% (n = 38) of residents. The relationships between the median cumulative discomfort score and individual resident or surgery characteristic in Phase 1 are shown in Table 2. Extremes of height, training seniority (Year 4 or 5), longer surgical duration (> 4 h) and operative complexity were found to be significant risk factors for greater physical discomfort.

**Table 2:** Cumulative physical discomfort scores in ESTEEMA Phase 1, Phase 2, and the difference in the scores from Phase 1 to 2.

Variable	Phase 1 (n = 46)		Phase 2 (n = 64)		Phases 1 & 2 differences
	Median cumulative physical discomfort score	*p* value	Median cumulative physical discomfort score	*p* value	*p* value
Gender		0.667		0.500	
Male	15 (n = 23)		13 (n = 31)		0.384
Female	16 (n = 23)		14 (n = 33)		0.603
Height (cm)		0.005		0.002	
≤ 160	32.5 (n = 2)		8 (n = 3)		*
161–170	17 (n = 26)		15 (n = 41)		0.509
171–180	13 (n = 17)		11.5 (n = 20)		0.992
> 180	39 (n = 1)		(n = 0)		*
Dominant hand		0.496		0.529	
Right	16 (n = 45)		14 (n = 63)		0.230
Left	12 (n = 1)		15 (n = 1)		*
Glove size		0.146		0.646	
< 7	17 (n = 31)		13 (n = 38)		0.121
≥ 7	13 (n = 15)		15 (n = 26)		0.509
Residency year		0.002		0.654	
R1–R3	14.5 (n = 38)		14 (n = 37)		0.741
R4–R5	27 (n = 8)		14 (n = 27)		0.013
Surgical role		0.470		0.992	
Cameraman	16 (n = 33)		14 (n = 40)		0.441
First assistant	17.5 (n = 13)		13.5 (n = 24)		0.418
Type of MAS		0.358		0.920	
Laparoscopy	16.5 (n = 44)		13.5 (n = 60)		0.465
Robotic	12 (n = 2)		14.5 (n = 4)		*
Duration (hours)		0.002		0.028	
< 4	13.5 (n = 36)		13 (n = 49)		0.728
≥ 4	21.5 (n = 10)		16 (n = 15)		0.020
Surgical specialty		0.086		0.227	
Upper GI	16.5 (n = 10)		8 (n = 8)		0.0007
HPB	20 (n = 12)		16 (n = 20)		0.250
Colorectal	12.5 (n = 24)		15 (n = 36)		0.478
Operative complexity		0.014		0.889	
Routine	13.5 (n = 28)		13 (n = 59)		0.711
Complicated	19 (n = 18)		14 (n = 5)		0.200

*p value not calculated because of small numbers of respondents in each arm.

**Figure 1:**
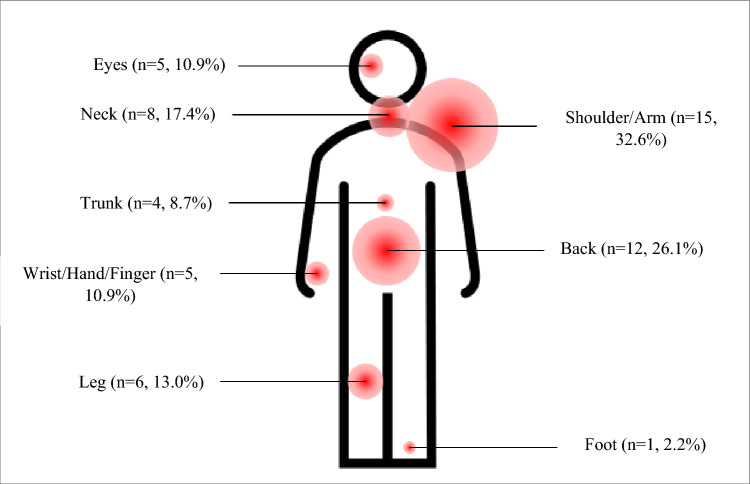
Body part distribution and frequencies of severe physical discomfort (defined as discomfort scores of 4 or 5) amongst 46 surgical trainees assisting minimally invasive abdominal surgery (ESTEEMA Phase 1).

The frequencies of severe physical discomfort (defined as discomfort scores of 4 or 5) across corresponding body parts are shown in Fig. 1. Over a third of respondents reported at a score of 4 or 5 in at least one body part (n = 17, 37.0%). Severe discomfort was most often reported in the shoulder or arm (1 in 3 residents), followed by the back (1 in 4 residents), and neck (almost 1 in 5 residents).

**Figure 2:**
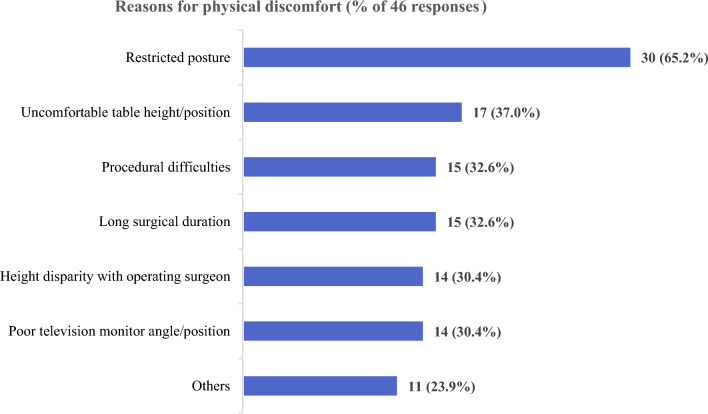
Frequencies of responses for factors contributing to surgical assistants’ physical discomfort during minimally invasive abdominal surgery (ESTEEMA Phase 1).

The frequencies of responses for intraoperative factors contributing to physical discomfort are provided in Fig. 2. The factor regarded as most contributory to discomfort during MAS surgery was the restricted posture (n = 30 out of 46 respondents, 65.2%), followed by uncomfortable table height or position (n = 17, 37.0%). Free text responses grouped under “Others” were unfamiliarity with the surgery (n = 5, 10.9%), unfamiliarity with surgical instruments (n = 3, 6.5%), uncomfortable gloves (n = 1, 2.2%), poor monitor resolution (n = 1, 2.2%), and dim lighting (n = 1, 2.2%).

**Figure 3:**
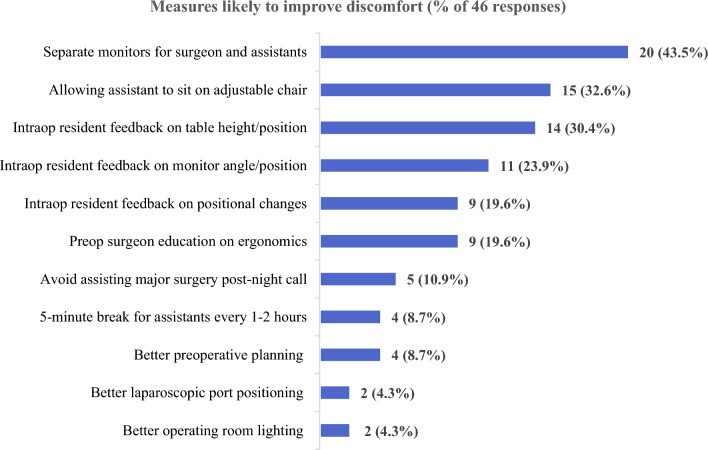
Frequencies of responses for measures likely to improve surgical assistants’ physical discomfort during minimally invasive abdominal surgery (ESTEEMA Phase 1).

The frequencies of responses for measures likely to improve physical discomfort during MAS surgery are shown in Fig. 3. The suggestion thought to be beneficial by most survey participants was separate monitors for the primary surgeon and the assistant (n = 20 out of 46 respondents, 43.5%), followed by allowing the surgical assistant to sit on an adjustable chair during surgery (n = 15, 32.6%), and intraoperative resident feedback or input concerning operating table height or position (n = 14, 30.4%). Besides the 6 suggested measures, free text responses provided by residents were preoperative surgeon education on ergonomics, avoidance of assisting surgery post-night call, better preoperative planning, better port positioning, and better operating room lighting.

### Phase 2 results

Sixty-four completed surveys were received during Phase 2 of the study. The relationships between the median cumulative discomfort score and resident or surgery characteristics during Phase 2 are shown in Table 2. Trainee height and a longer operative duration were significant risk factors for physical discomfort in Phase 2 (Table 2). Of 18 attending MAS surgeons across the three institutions who were informed of the study, 14 (77.8%) agreed to administer the ergonomic improvement measures intraoperatively when possible. Following introduction of intraoperative measures to improve ergonomics, the overall rate of MAS assistants with physical symptoms and severe discomfort improved to 81.3% (n = 52) from 82.6% and 34.4% (n = 22) from 37.0% respectively, although these differences were not statistically significant.

**Figure 4:**
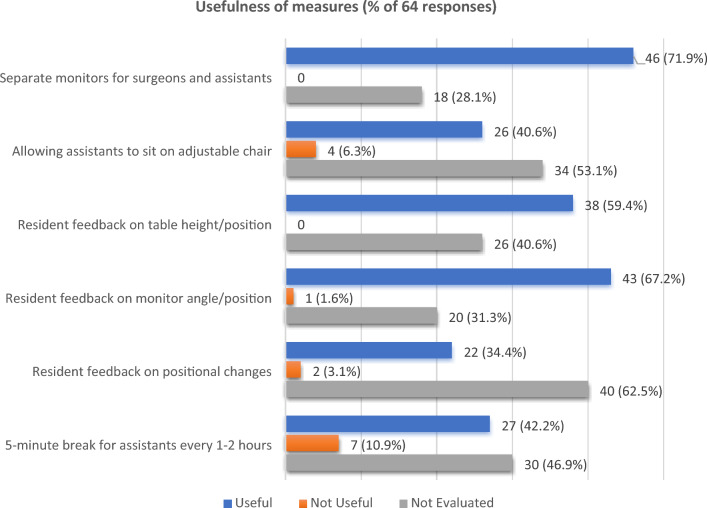
Evaluation of measures used to improve surgical assistants’ physical discomfort during minimally invasive abdominal surgery (ESTEEMA Phase 2).

Of the free text options provided by residents for improving ergonomics, most pertained to optimizing preoperative planning or raising awareness of assistants’ ergonomic challenges, rather than additional intraoperative measures which could be undertaken to reduce physical symptoms. As decided by the study team, the Phase 2 survey therefore only evaluated the original 6 suggested measures to reduce assistant discomfort. The results of these are provided in Fig. 4.

The evaluated measure which was most found useful was having separate television monitors for the primary surgeon and assistants (n = 46 out of 64 respondents, 71.9%), followed by intraoperative resident feedback on the television monitor angle or position (n = 43, 67.2%), and subsequent adjustment of the screen. The evaluated measure which was most found ineffective was a 5-min break for the assistant every 1–2 h of surgery (n = 7, 10.9%), followed by having an adjustable chair to sit down on (n = 4, 6.3%). The measure which was least evaluated was resident feedback during the surgery on the need for self-adjustment of position due to physical discomfort (n = 40, 62.5%), followed by having a chair to sit down (n = 34, 53.1%).

## Discussion

Surgeons are well known for their confidence and composure in the face of adversity^24^. In the operating room, seasoned surgeons have acquired the reputation of being masters of endurance, tolerating long hours of surgery with associated mental strain and physical fatigue without voicing complaint^25^. These characteristics are often inculcated during training years, where resilience and perseverance are qualities often thought essential for overcoming the rigors of surgical qualification.

Workplace guidelines have been introduced to balance the need for adequate surgical training with resident welfare and patient safety^26,27^. While these guidelines impose working hour limits and mandatory days off, the effects of long years of surgical assistance have yet to be adequately addressed. This is particularly relevant considering the minimally invasive revolution over the recent decades heralding an “impending epidemic” of physical disorders amongst laparoscopic surgeons and their operative assistants^15^. A recent meta-analysis showed that 12% of proceduralists who suffer a work-related musculoskeletal disorder required a leave of absence, practice restriction, modification, or early retirement^28^.

As anticipated, complex surgery and prolonged operative duration of greater than 4 h significantly increased MAS assistants’ physical symptoms (Phase 1). Residents in their 4th and 5th years of training also experienced greater discomfort than those within their first three years. This may reflect the complexity of surgery that more experienced trainees are required to assist with (50% of cases assisted by Year 4 or 5 residents in Phase 1 were complex or had operative time > 4 h). Another explanation involves the accumulation of physical ailments from junior to senior residency. Extremes of height was also shown to be a risk factor for increased physical discomfort; however, only one resident above 180 cm and only two below 160 cm were in the Phase 1 respondent cohort.

Following introduction of intraoperative measures to improve ergonomics in Phase 2, resident seniority and operative complexity ceased to be significant risk factors for physical discomfort. There was also a general trend of improvement in assistant self-reported discomfort across most resident and surgery characteristics, although this was not statistically significant.

Previous studies showed that women are at greater risk of physical discomfort during MAS surgery compared to men^29,30^, with Wong et al. demonstrating a fivefold increase in the chances of physical complaints amongst women^30^. A smaller glove size has also been reported to significantly increase difficulty using laparoscopic devices^19,31^. Both female gender and a smaller glove size were not found to be risk factors for physical symptoms amongst our study cohort. This is likely related to the fact that operative assistants, unlike primary surgeons, are not required to consistently manipulate laparoscopic instruments such as energy devices and surgical staplers.

None of the “free text” suggestions listed by Phase 1 respondents for improving ergonomics were evaluated in Phase 2, for reasons discussed in the Results section. However, close to 20% of survey respondents felt that preoperative surgeon education on operative ergonomics was likely to improve physical discomfort. This may reflect the perception amongst surgical trainees that primary surgeons are unaware of or do not prioritize surgical assistant ergonomics. A previous survey of 83 surgeons showed that greater than two thirds were unaware of possible ergonomic solutions and did not consider adopting any preventive methods^32^.

Our study is limited by the relatively small number of completed responses, as a proportion of residents were rotating through non-abdominal postings or assisting open surgery during the study duration. Several categories had very few respondents, including extremes of height, left-handedness, or robotic surgery. The results associated with these categories should be interpreted with caution. The robotic-assisted approach was not routine for most MAS procedures at our unit. Moreover, a camera operator is unnecessary for robotic surgery, further reducing the number of trainee responses.

As the surveys were anonymous, it was not possible to determine the exact number of unique respondents per study phase. Resident responsibilities may change intraoperatively, with residents often alternating between camera operator, first assistant, and even the main surgeon during different periods of the same surgery. Nonetheless, the survey allowed resident assistants to select what they perceived to be their primary role during MAS surgery.

In addition, there was considerable differences between respondent characteristics, as well as type and perceived complexity of cases between Phase 1 and 2 of the study. While this represents the real-world diversity of trainee profiles and operative encounters, comparability of the results between the two phases may be limited.

Heterogeneity of primary surgeon preferences, departmental practices, and operating room facilities across institutions and subspecialty departments would have influenced assistant fatigue and the ability to assess ergonomic improvement proposals. For example, it was already routine practice in one institution for camera operators to adopt a seated position during laparoscopic hepatobiliary surgery. Multiple adjustable ceiling mounted television monitors were also available in certain operating rooms but not in others.

Despite these limitations, this study reflects the results from the largest healthcare cluster in Singapore with the biggest pool of general surgery residents. To our knowledge, it is also one of the only studies assessing MAS assistants’ physical discomfort in an Asian setting. Unlike previous reports, a strength of this study is the use of a 5-point Likert scale for physical discomfort instead of a dichotomous yes or no, allowing more nuance in symptom grading.

Our broad-based study serves as platform upon which further areas of in-depth analysis can be performed for specific subspecialty areas or trainee cohorts. Objective measures of muscle fatigue, such as electromyography recordings, can be performed to circumvent the subjectivity of self-reported data^33^. The inclusion of more holistic pre- and postoperative measures, including specified exercises or muscle therapies, may also be beneficial. Assessment of attending surgeon awareness and perspectives will better define the long-term feasibility of proposed ergonomic measures and help to overcome the appreciable challenges during their implementation. The effect of formal training in ergonomics for both the primary surgeon and operative assistants should be evaluated.

## Conclusion

Physical discomfort is prevalent amongst surgical trainees who assist MAS surgery. Knowledge of ergonomic risk factors and mitigating measures can improve trainees’ well-being and potentially prolong career longevity.

## Data Availability

The datasets used and analyzed for the current study are available from the corresponding author upon reasonable request.
